# Interactions Between Zinc and Thymulin

**DOI:** 10.1155/MBD.1994.233

**Published:** 1994

**Authors:** Mireille Dardenne, Jean-Marie Pleau

**Affiliations:** 1 CNRS URA 1461 Hôpital Necker 161, rue de Sèvres Paris F - 75015 France

## Abstract

Thymulin (formerly called "Facteur Thymique Sérique or FTS) is a metallopeptidic hormone selectively produced by thymic epithelial cells (TEC) and known to induce intra and extra-thymic T cell differentiation. It was initially isolated from porcine serum and shown to be present in calf thymus extract. Its amino-acid sequence was determined (<Glu-Ala-Lys-Ser-Gln-Gly-Gly-Ser-Asn). It is a nonapeptide whose biological activity is dependent on the presence of zinc, in an equimolecular ratio. The metallopeptide thus formed bears a specific tridimensional conformation detected by nuclear magnetic resonance studies, and that yielded a new monoclonal antibody-defined epitope. The presence of zinc and metallothionein has been demonstrated within TEC which produce the peptide, suggesting that the molecule is secreted in its active zinc-containing form. The zinc/thymulin relationship, was further studied using various models of mild zinc deficiency in experimental animals and in humans. Serum thymulin activity was decreased as a result of zinc deficiency, and was corrected by in vivo and in vitro zinc supplementation, suggesting that this parameter could be a sensitive indicator of zinc deficiency. When considered together with the parallel variations seen in T-cell subpopulations and lymphokine production, these observations could provide a possible explanation of the role of zinc on T cell functions.


### INTERACTIONS BETWEEN ZINC AND THYMULIN

Mireille Dardenne* and Jean-Marie Pleau

CNRS URA 1461, Hôpital Necker, 161, rue de Sèvres, F - 75015 Paris, France

### 1. ABSTRACT

Thymulin (formerly called "Facteur Thymique Sérique or FTS) is a metallopeptidic hormone selectively produced by thymic epithelial cells (TEC) and known to induce intra and extra-thymic T cell differentiation. It was initially isolated.from porcine serum and shown to be present in calf thymus extract. Its amino-acid sequence was determined (<Glu-Ala-Lys-Ser-Gln-Gly-Gly-Ser-Asn). It is a nonapeptide whose biological activity is dependent on the presence of zinc, in an equimolecular ratio. The metallopeptide thus formed bears a specific tridimensional conformation detected by nuclear magnetic resonance studies, and that yielded a new monoclonal antibody-defined epitope. The presence of zinc and metallothionein has been demonstrated within TEC which produce the peptide, suggesting that the molecule is secreted in its active zinc-containing form. The zinc/thymulin relationship, was further studied using various models of mild zinc deficiency in experimental animals and in humans. Serum thymulin activity was decreased as a result of zinc deficiency, and was corrected by in vivo and in vitro zinc supplementation, suggesting that this parameter could be a sensitive indicator of zinc deficiency. When considered together with the parallel variations seen in T-cell subpopulations and lymphokine production, these observations could provide a possible explanation of the role of zinc on T cell functions.

### 2. INTRODUCTION

It is now widely accepted that the trace element zinc exerts a powerful and apparently specific influence on the thymus, on T lymphocytes and on cellular immunity, resulting in a strong immune modulatory activity on cell-mediated responses, in both human and animal systems (1,2).

It is apparent that zinc deficiency severely depresses immune responses. Experimental zinc deprivation (with all other nutritional elements at a normal level) leads to generalized lymphoid hypoplasia, rapid thymic involution, particularly of the cortex, decreased production of thymic hormones, impaired lymphocyte proliferative capacity after phytomitogen stimulation and reduced antibody and cell-mediated responses.

It thus appears quite clear that zinc deficiency has multiple consequences on the immune system and that they are similar to those observed after thymectomy, either neonatal thymectomy in extreme cases of deficiency where zinc deprivation is total and occurs very early, or thymectomy in the young adult when deficiency is induced during adulthood (3).

Various mechanisms can explain the early thymic atrophy observed in zinc-deprived mice. Rapidly replicating lymphocytes in the thymus could be particularly sensitive to the functional anomaly of zinc-rich metalloenzymes required for lymphocyte proliferation. Another hypothesis is that the epithelial function of the thymus could be specifically altered (4,5). A third involves direct action of thymulin, a thymic hormone molecule. Several authors have reported that the serum level of thymulin was abnormally decreased in zinc deficiency, both in the mouse and in man (5,6).

Data from our laboratory favored the view that thymulin was directly dependent on zinc, a finding which could explain many of the observations reported above. In the present review, we shall summarize the data which have appeared in the last few years concerning the relationship between zinc and thymulin analysed both biochemically and biologically, as well as the alteraction occurring in some animal and human zinc deficiencies.

### 3. ZINC/THYMULIN INTERACTIONS

### 3.1. Biochemical aspects

Thymulin is a nonapeptide produced by the thymic epithelium originally isolated from serum, hence its former name, "facteur thymique sérique" (7,8). It induces or enhances the expression of a number of T-cell markers and functions, notably the Thy-1 differentiation antigen (9).

The aminoacid sequence of this peptide was established (Pyro-Glu-Ala-Lys-Ser-Gln-Gly-Gly-Ser-Asn), and on the basis of this sequence a biologically active synthetic peptide was obtained (10). However, some synthetic preparations were surprisingly unstable or inactive, thus suggesting the need for a posttranslational phenomenon in the expression of the biologically active molecule. Actually, a number of findings described below demonstrated that the binding of some metals and particularly zinc is implicated in the biological activity of thymulin.

Several approaches were developed in our laboratory to seek the presence of a transition metal in the biologically active form of thymulin.

3.1.1. Effect of chelex treatment and metal salt addition upon the biological activity of thymulin

We first observed that synthetic or natural thymulin lost its biological activity after treatment with a chelating agent such as chelex 100, and that this activity was completely restored by the addition of $ZnCl_2$ or to a lesser extent, of some other metals (galium, aluminium and copper). Moreover, we noted that the highest degree of activation was achieved when thymulin was mixed with zinc in an equimolecular ratio. These experiments thus represented the first evidence for the existence of a thymulin-metal complex. This interaction was further confirmed by gel chromatography using a mixture of radioactively labeled thymulin and $ZnCl_2$ (11).

3.1.2. Physicochemical parameters for zinc-thymulin binding

The affinity, the numbers of binding sites and the relative specificity of zinc binding to thymulin were established by equilibrium gel chromatography (12). We demonstrated that at pH 7.5 thymulin presented 1 ± 0.2 binding site for zinc with an apparent dissociation constant in the order of $5 \pm 2.10^{-7}$M.

Interestingly, at this same pH, some other metals, particularly gallium and aluminium, competed with zinc for binding to thymulin, actually conferring biological activity to the peptide. In addition, structural analogues of thymulin were used to determine which aminoacids were present in the zinc-binding site of thymulin. The data obtained allowed us to establish the importance of asparagine, and the role of a particular peptidic bound (between the aminoacid residues 5 and 6) in thymulin sequence. As detailed below, these findings were confirmed and extended by nuclear magnetic resonance (NMR) studies.

3.1.3. Conformational characteristics of thymulin determined by NMR

Preliminary studies were performed on the metal free peptide (in DMSO solution and in aqueous medium) using one or two dimensional $^1H$, $^{13}C$ NMR and circular dichroism . These studies strongly suggested that the free nonapeptide in aqueous solution is flexible and in rapid equilibrum between multiple conformations. In DMSO-$d_6$, thymulin adopted a partially folded conformation stabilized by three intramolecular hydrogen bounds (13). In view of these results, similar studies were performed on the thymulin-zinc complex. These experiments clearly indicated the existence of two different complexes associating zinc and thymulin, in respective ratios of 1:1 and 1:2. Moreover, we showed that, for the 1:1 complex, the Asn[9] as well as the Ser[4] and [8] residues were involved in zinc binding (Figure 1). These results were confirmed by the analysis of some thymulin analogues(14). Since as mentioned above, the complexes containing aluminium, gallium and to a lesser extent copper have proved to be biologically active, studies of other metal complexes of the nonapeptide were performed. These studies demonstrated that the changes induced by the addition of zinc were quite different from those observed with other metal salts suggesting that the coordination of each ion induced a unique conformation (15,16).

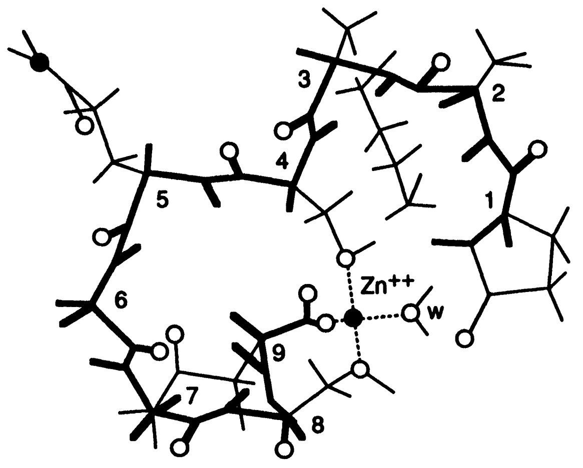

Fig. 1 Schematic representation of the 1:1 zinc-nonapeptide complex showing the Ser[4]-OH, Ser[8]-OH and Asn[9]-$CO_2$ sites coordinated to the metal ions.

3.1.4. Zinc-dependent epitope on the molecule of thymulin

In the past few years, we produced monoclonal antibodies (MAb) directed against synthetic or natural (intracellular) thymulin (17,18). Both kinds of antithymulin MAb were screened for their ability : a) to inhibit the activity of synthetic or seric thymulin in the rosette assay routinely used to measure thymulin biological activity, and b) to bind specifically to thymic epithelial cells (TEC) in an immunofluorescence assay.

More recently, we observed that these MAb recognized the thymulin molecule only if it was coupled with zinc (19). Thus, for example, when synthetic or seric thymulin was subjected to a chelation procedure, it was no longer detectable by the antibodies in either the rosette or the immunofluorescence assay.

However, when zinc was added in an equimolecular ratio to the chelated peptide, the anti-thymulin MAb were again able to bind to the hormone. These data confirmed that the presence of zinc in the molecule of thymulin determines a spatial conformation which, in addition to being necessary to its biological activity, yields a new epitope that can be specifically recognized by monoclonal antibodies. This epitope is strictly dependent upon the presence of zinc and not of other metals, such as Al or Cu, which are able to confer the biological activity to the peptide but form with it complexes not recognized by the monoclonal antibodies (19). These comparative studies suggested that the 1:1 zinc-thymulin complex was responsible for the specific recognition of the thymic hormone by monoclonal antibodies.

### 3.2. Demonstration of the presence of zinc within the thymus

Direct evidence for the presence of zinc within the thymus has come from our previous observations using electron microprobe analysis on ultrathin sections of mouse thymus. This technique allowed us to detect zinc inside cytoplasmic vacuoles of TEC, and further experiments demonstrated that this metal can be accumulated within these subcellular structures in thymuses from mice previously injected with zinc chloride (20). These vacuoles were also shown to contain thymulin.

These data clearly indicated that zinc could be stored within TEC by some specific mechanism. One hypothetical candidate for the role in the intra-thymic storage of zinc could be metallothionein. This low molecular weight protein has a high cysteine content and is known to bind, with high avidity, class II B transitional metals, namely zinc, cadmium and copper (21). Moreover, it had been previously shown that it is highly expressed in cells that can store zinc (22). These findings led us to look for metallothionein within the thymus, and we demonstrated by immunocytochemical means the presence of this protein in TEC both in vivo and in vitro (23). A closer relationship between metallothionein, zinc and thymulin was established by in vitro experiments showing that zinc-loaded metallothionein released zinc which was eventually trapped by thymulin. In this respect our findings support the notion that metallothionein can function as a zinc acceptor/donor.

### 4. ZINC DEFICIENCY, ZINC SUPPLEMENTATION AND THYMULIN

### 4.1. Experimental zinc deprivation

The zinc/thymulin relationship was further investigated in our laboratory using two models of in vivo zinc deprivation. First, active thymulin levels were studied in sera from mice subjected to a long-term, marginally zinc-deficient diet. Despite the absence of thymic atrophy, a significant decrease in the serum levels of thymulin was observed as early as two months after beginning the diet. However, these levels were consistently restored after in vitro addition of ZnCl2 (5-6). These findings strongly suggest that the non-active zinc-deprived peptide is secreted in these experimental conditions. Interestingly, analysis of thymuses from Zn-deprived mice showed that, in spite of the absence of major changes in thymic epithelial cells, there was a progressive increase in the number of thymulin-containing cells, suggesting an increase in the production of the hormone. These observations indicate a compensatory phenomenon, similar to the classical feedback response already described for thymulin secretion (24). In the case of marginal Zn deficiency, this feedback effect could be stimulated by the decline of the hormone in its active form, suggesting that the biological regulatory system recognizes only the peptide containing zinc. This hypothesis is in agreement with the fact that all the thymulin functions investigated to date require the presence of zinc in the molecule.

More recently, we made similar observations in human volunteers submitted to zinc-deficient diets. Their thymulin levels, which were in a normal range at the beginning of the diet, dropped very rapidly during zinc deprivation, in close parallel with zinc plasma levels, and were restored to normal after in vivo zinc repletion. In addition, the low thymulin levels observed during zinc deprivation were also restored to normal values by in vitro addition of zinc chloride (25). These results represent a further argument in favor of natural coexistence of the active (zinc containing) and inactive (zinc-deprived) forms of thymulin in the serum, the latter being predominant in zinc-deficient conditions.

### 4.2. Effect of oral zinc supplementation in aging mice

The physiological age-dependent decline of thymulin serum levels has already been extensively described (26,27). As we first demonstrated in 1972, the serum level of this hormone is maximal at birth, remains constant until the age of 15 to 20 years and then declines progressively, a finding which has been confirmed by other investigators (28,29). This phenomenon does not seem to be restricted to thymulin. Accumulating evidence shows that the circulating levels of other well defined thymic hormones such a thymopoietin and thymosin $\alpha 1$ also decrease as a function of age.

The studies of thymuses from mice of different ages showed that in normal animals the age-dependent decline of thymulin in the serum is paralleled by a decrease in the number of thymulin containing cells, measured by immunofluorescence with anti-thymulin monoclonal antibodies (27). These data indicated that the diminished levels of circulating thymulin observed in aging are due, at least partially, to a primary decreased production of the hormone within the thymus.

However, some recent observations suggested that age-related thymic dysfunction and in particular thymic hypotrophy were not totally irreversible phenomena but could be secondary to environmental alterations such as zinc deficiency.

We and other groups have been prompted to study the effect of oral zinc supplementation on some immune parameters in old mice. On the basis of findings that a critical trace element, zinc, is required for a considerable number of physiological functions in differentiation and proliferation of cell lineages and synthesis and release of molecules with enzymatic and hormonal action, and that modification of zinc turnover has been reported to occur with advancing age. Muzzuioli et al. (30) showed that zinc supplementation in old mice induced regrowth of thymic tissue with reenlargment of the cortex and recovery of its endocrine activity as measured by the plasma level of thymulin, and by the number of thymic hormone secreting cells in thymic medulla. At the peripheral level an increased proportion of Thy 1.2+ and Lyt1+ cells in the spleen and partial recovery of PHA response and NK activity was observed after zinc supplementation.

Similar data on immune parameters were obtained in our laboratory (31). These findings suggest that the age dependent involution of the thymus is largely dependent on the progressive loss of zinc bioavaibility that occurs which advancing age.

### 4.3. Zinc deficiency in pathological situations

We and other groups recently studied thymulin levels in various pathological situations with zinc deficiency : the first study was performed in young children with nephrotic syndrome where we observed low thymulin levels, compared to age-matched subjects. These low levels of thymulin activity were restored to normal after remission of the disease or after in vitro addition of zinc salts to the serum under study (32). In addition, we performed similar studies in subjects with sickle cell anemia. Studies have shown that several parameters of cellular immune function may be altered in such patients and are related to a zinc deficiency. In these patients, thymulin levels were found to be significantly lower than in age-matched healthy subjects. However, as previously observed in human volunteers in whom restricted zinc intake induced a mild specific deficiency of zinc, paralleled with low thymulin levels, we observed that zinc supplementation or in vitro zinc activation restored normal thymulin values (25). Similar results have been recently reported in patients with chronic renal failure and zinc deficiency in which zinc therapy significantly increased zinc-bound thymulin in children with Down's syndrome and in patients with type-I diabetes (33-35).

The mechanism of action of zinc in vivo in these various situations is still unclear. In particular, it is still open to question whether zinc simply activates pre-existing thymulin molecules, as shown by in vitro studies, or whether it might also increase the production of thymulin by thymic epithelial cells.

Nevertheless these observations that zinc is required to render thymulin biologically active suggest that functional thymulin deficiency could contribute significantly to the immune deficiency induced by lack of zinc intake.

### 5. CONCLUSIONS

The bulk of data reported in the last few years and summarized above strongly indicate that at least part of the multifaceted intrathymic functions of the trace element zinc may occur via the activation of the thymic hormone thymulin.

As judged by our findings, the zinc/thymulin coupling probably takes place before the hormone release by thymic epithelial cells. In this context, the mature (zinc-containing) form of the hormone would be able, immediately after being secreted, to influence surrounding thymocytes through its zinc-dependent active site.

### REFERENCES

1 Bach JF: *Immunol Today* **2**:225, 1981
2 Prasad AS: *Clinics in Endocrinol and Metab* **14**:567, 1985
3 Nash L, Iwata T, Fernandes G, Good PA, Incefy G: *Cell Immunol* **48**:238, 1979
4 Chandra RK, Heresi G, Au B: *Clin Exp Immunol* **42**:332, 1980
5 Iwata T, Incefy GS, Menendez-Botet TG, Phi K, Good RA: *Cell Immunol* **47**:100, 1983
6 Dardenne M, Savino W, Wade S, Kaiserlian D, Lemonnier D, Bach JF: *Eur J Immunol* **47**:454, 1984
7 Dardenne M, Pleau JM, Man NK, Bach JF: *J Biol Chem* **252**:8045, 1977
8 Pleau JM, Dardenne M, Blouquit Y, Bach JF: *J Biol Chem* **252**:8040, 1977
9 Bach JF: *Clinical Immunology and Allergy* **3**:1, 1983
10 Bach JF, Dardenne M, Pleau JM, Rosa J: *Nature* **266**:55, 1977
11 Dardenne M, Pleau JM, Nabarra B, Lefrancier P, Derrien M, Choay J, Bach JF: *Proc Natl Acad Sci USA* **79**:5370, 1982
12 Gastinel LN, Dardenne M, Pleau JM, Bach JF: *Biochim Biophys Acta* **797**:147, 1984
13 Laussac JP, Cung MT, Pasdeloup M, Haran H, Marraud M, Lefrancier P, Dardenne M, Bach JF: *J Biol Chem* **261**:7784, 1986
14 Cung MT, Marraud M, Lefrancier P, Dardenne M, Bach JF, Laussac JP: *J Biol Chem* **263**:5574, 1988
15 Laussac JP, Pasdeloup M, Lefrancier P, Dardenne M, Bach JF: *New J Chem* **11**:67, 1987
16 Laussac JP, Lefrancier P, Dardenne M, Bach JF, Marraud M, Cung MT: *Am Chem Soc* **27**:4094, 1988
17 Dardenne M, Pleau JM, Savino W, Bach JF: *Immunol Letters* **4**:79, 1982
18 Berrih S, Savino W, Azoulay M, Dardenne M, Bach JF: *J Histochem Cytochem* **32**:432, 1984
19 Dardenne M, Savino W, Berrih S, Bach JF: *Proc Natl Acad Sci USA* **82**:7035, 1985
20 Nabarra B, Halpern S, Kaiserlian D, Dardenne M : *Cell Tissue Res* **238**:209, 1984
21 Kaga JH, Kohima Y, Kisslng MM, Leich K: *Ciba Foundation Symp* **72**:223, 1980
22. Danielan KJ, Ohi S, Hayang PC: *Proc Natl Acad Sci USA* **79**:2301, 1982
23 Savino W, Huang PC, Corrigan A, Berrih S, Dardenne M: *J Histochem Cytochem* **32**:942, 1984
24 Savino W, Dardenne M, Bach JF: *Clin Exp Immunol* **52**:7, 1983
25 Prasad S, Meftah S, Abdallah J, Kaplan J, Brewer GJ, Bach JF, Dardenne M: *J Clin Invest* **82**:1202, 1988
26 Bach JF, Dardenne M, Papiernik M, Barois A, Levasseur P, Lebrigand H: *Lancet* **2**:1056, 1972

27 Savino W, Dardenne M, Bach JF: *Clin Exp Immunol* **52**:1, 1983
28 Fabris N, Mocchegiani E: *Cell Immunol* **91**:325, 1985
29 Iwata T, Incefy GS, Menendez-Botet TG, Phi K, Good RA: *Cell Immunol* **47**:100, 1983
30 Muzziolo M, Mocchegiani E, Fabris N:Zinc modulates neuroendocrine immune interactions in aged mice.
32 Bensman A, Dardenne M, Morgant C, Vasmant D, Bach JF: *Int J Pediatric Nephrol* :201, 1983
33 Travaglini P, Moriondo P, Togni E, Benegoni P, Bochicchio D, Conti A, Ambroso G, Ponticelli C, Mocchegiani E, Fabris N, Faglia G: *J Clin Endocrin Metab* **68**:186, 1989
34 Franceschi C, Chiricolo M, Licastro F, Zannotti M, Masi M, Mocchegiani E, Fabris N: *J Mental Deficiency Res* **32**:169, 1988
35 Mocchegiani E, Boemi M, Fumelli P, Fabris N: *Diabetes* **12**:932, 1989

**Received: September 29, 1993**

